# Metatranscriptome analysis of fungal strains *Penicillium camemberti* and *Geotrichum candidum* reveal cheese matrix breakdown and potential development of sensory properties of ripened Camembert-type cheese

**DOI:** 10.1186/1471-2164-15-235

**Published:** 2014-03-26

**Authors:** Marie-Hélène Lessard, Catherine Viel, Brian Boyle, Daniel St-Gelais, Steve Labrie

**Affiliations:** 1 Department of Food Sciences and Nutrition, Institute of Nutrition and Functional Foods (INAF), STELA Dairy Research Centre, Université Laval, 2425 rue de l′Agriculture, G1V 0A6, Québec City, QC, Canada; 2 Institut de Biologie Intégrative et des Systèmes (IBIS), Pavillon Charles-Eugène-Marchand, 1030, rue de la Médecine, Université Laval, G1V 0A6, Québec City, QC, Canada; 3 Agriculture and Agri-Food Canada, Food Research and Development Centre, 3600 Casavant Blvd. West, Saint Hyacinthe J2S 8E3 QC, Canada

## Abstract

**Background:**

Camembert-type cheese ripening is driven mainly by fungal microflora including *Geotrichum candidum* and *Penicillium camemberti*. These species are major contributors to the texture and flavour of typical bloomy rind cheeses. Biochemical studies showed that *G. candidum* reduces bitterness, enhances sulphur flavors through amino acid catabolism and has an impact on rind texture, firmness and thickness, while *P. camemberti* is responsible for the white and bloomy aspect of the rind, and produces enzymes involved in proteolysis and lipolysis activities. However, very little is known about the genetic determinants that code for these activities and their expression profile over time during the ripening process.

**Results:**

The metatranscriptome of an industrial Canadian Camembert-type cheese was studied at seven different sampling days over 77 days of ripening. A database called *CamemBank*01was generated, containing a total of 1,060,019 sequence tags (reads) assembled in 7916 contigs. Sequence analysis revealed that 57% of the contigs could be affiliated to molds, 16% originated from yeasts, and 27% could not be identified. According to the functional annotation performed, the predominant processes during Camembert ripening include gene expression, energy-, carbohydrate-, organic acid-, lipid- and protein- metabolic processes, cell growth, and response to different stresses. Relative expression data showed that these functions occurred mostly in the first two weeks of the ripening period.

**Conclusions:**

These data provide further advances in our knowledge about the biological activities of the dominant ripening microflora of Camembert cheese and will help select biological markers to improve cheese quality assessment.

## Background

Camembert cheese is a soft, mold-ripened cheese. The mold *Penicillium camemberti* and the yeast *Geotrichum candidum* are the two major *Fungi* that give the white coated characteristic of this cheese variety. Their association is crucial not only for appearance, but also for typical sensory characteristics of Camembert cheese [1,2]. Previous studies considered aroma production in pure culture, on culture media or on model cheese medium [3], biochemical pathways potentially involved in the development of sensory properties and even microbiological succession during Camembert ripening [4-10]. Surprisingly, only limited genetic information is available for these *Fungi*, since fewer than 30 different genes of each organism have been deposited in public databases.

Molecular biology techniques were recently used to evaluate several aspects of sensory characteristics of cheese. For example, multispecies DNA microarrays combined with biochemical analysis (HPLC and SPME-GCMS) has been a useful tool to evaluate L-methionine catabolism, production of volatile sulfur compounds (VSC) and lactose/lactate consumption during yeast growth [11,12]. Even though microarrays provide information about gene expression under various conditions, their utility is limited to organisms for which genetic information is available [13]. Next-Generation sequencing (NGS) methods are now widely used for *de novo-* and re- sequencing of genomes, transcriptomes, epigenomes and metagenomes [14-19]. The first metagenomic analysis using 454 pyrosequencing was performed on bacterial communities in mines [20] and since then, high quality information is available about ecosystems from soil [21,22], sea water [23,24], humans [25,26], and even cheese [27], most of them identifying microorganisms and establishing their phylogenetic relationships [28]. Genome and metagenome sequencing are powerful tools, but massive transcriptome sequencing using NGS provides a more dynamic and functional view of microbial activity under particular conditions by accumulating data on RNA and its expression profile.

Several studies used NGS technologies to compare the transcriptomic response of a single organism exposed to different conditions [29-35]. In multiple-organism environments, establishing the metatranscriptome reveals the activity of a community, but only rare and very recent papers selected this approach [36-39]. This study is the first comprehensive metatranscriptome analysis of the Camembert cheese complex fungal ripening ecosystem. Here, the fungal metatranscriptome was sequenced using a Roche 454 pyrosequencing NGS strategy, without prior knowledge of the *Penicillium camemberti* and *Geotrichum candidum* genome sequences. The longer reads produced by the 454 instruments enabled the discovery and characterization of new genetic information for these *Fungi* and simultaneously established their activity profile. Many fungal activities were identified using this strategy, including the central metabolism and the response to environmental stresses and nutrient availability in the cheese matrix. This semi-quantitative gene expression profiling revealed the adaptation of *G. candidum* and *P. camemberti* during the 77-day ripening period of a commercial Canadian Camembert-type cheese.

## Results and discussion

### Cheese characteristics and fungal growth

**Figure 1:**
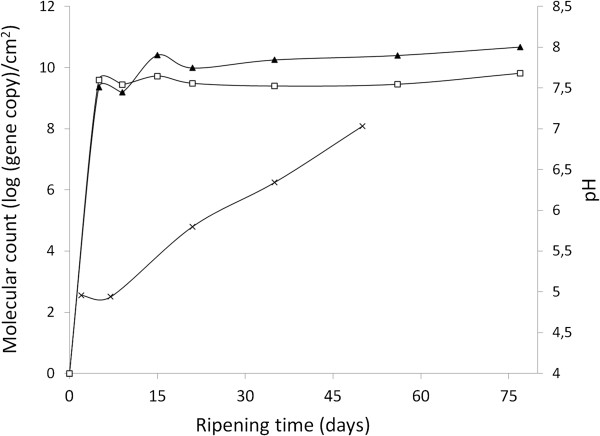
**Evolution of pH and fungal growth during Camembert cheese ripening.** The ripening culture was a mixture of (□) *G. candidum* LMA-1028 and (▲) *P. camemberti* LMA- 1029. Each strain was quantified individually using a TaqMan real-time qPCR method [41], over 77 days of ripening. pH (×) measures were taken weekly until day 50.

Commercial Camembert-type cheeses made from pasteurized milk were obtained from a processing plant located in Canada. Cheeses used in the present study developed no obvious defects during the ripening period and met the high quality criteria of the company who provided the cheeses for the characteristics of cheese texture, fat matter, salt and water content (confidential data, not presented). Also, the measured pH increase fit the normal alkalinisation of the rind over time observed for similar Canadian mold ripened cheeses (Figure 1) [40]. When fungal strains selected for this cheese were quantified using a TaqMan-based qPCR method [5,41], *G. candidum* and *P. camemberti* had similar growth profiles with an active phase in the first 5 days of ripening. Their maximum cell density was 6.45 × 10^9^ and 4.69 × 10^10^ gene copies/cm^2^, respectively, at the end of ripening (Figure 1).

### Sequencing and assembly of the Camembert cheese transcriptome

Since only scarce genetic information is available for *G. candidum* and *P. camemberti*, the metatranscriptomic approach using massive parallel sequencing had the advantage of simultaneously identifying new genes and determining their expression profile during cheese ripening. A *de novo* assembly performed using all 1,019,060 reads generated 8,909 contigs (length > 99 nt, average length of 916 nt). After sorting data for a minimum contig length of 200 nt and a minimum of 6 assembled reads, 8,318 contigs were conserved in the original cheese database. Reads were mapped back to the *de novo* assembly to enable semi-quantitative analysis and quality control of the assembly. *De novo* assembly and mapping data were compared to remove artefacts, such as duplicated transcript models, resulting in the exclusion of 402/8,318 contigs. The assembly contigs were free of fungal rDNA and mt-rDNA contamination as revealed by local BLAST search. This high quality dataset of 7,916 contigs (average length of 988 nt; Table 1) represents the fungal metatranscriptome of the Canadian Camembert-type cheese selected and was called *CamemBank*01, henceforth compensating the absence of available sequenced genomes for ripening species *Penicillium camemberti* and *Geotrichum candidum*.

**Table 1:** **Sequencing statistics and expression data in *CamemBank*** **01**

**Total number of reads in *CamemBank*** **01**	**1**,**019**,**060 reads**
Total number of filtered contigs in *CamemBank*01	7916 contigs
Average length per contig	988 bp
Minimum length	202 bp
Maximum length	4994 bp
Average expression per contig	71 reads/contig
Minimum expression	6 reads/contig
Maximum expression	10,928 reads/contig
Number of contigs with an expression	
< 71 reads/contig	6644 contigs (84%)
≥ 71 reads/contig	1272 contigs (16%)

### Identification and functional annotation of contigs found in *CamemBank*01

All 7,916 contigs were analyzed using the Blast2GO platform [42]. Because no genome of the yeast *G. candidum* and the mold *P. camemberti* are currently available in public databases, sequence analysis was performed with caution. Therefore, contigs were assigned according to their similarity to mold or yeast relatives if sequences had a >70% identity with known proteins in GenBank. Globally, 56,7% contigs originated from molds (M, n = 4,491 contigs) and 16,4% from yeasts (Y, n = 1,299 contigs). The other 26,9% was defined as of uncharacterized origin (U), either because the Blastx protein similarity was under 70% or because they had no significant homology. Over the 563,733 reads assembled, 275,586 reads (48.89%) were confidently assigned to molds and 105,017 reads (18.63%) to yeasts, while 183,130 reads are still unassigned. The average expression was 71 reads/contig, or 71 transcripts/gene (Table 1). At each sampling time, the majority of expressed contigs originated from molds and the average proportions of M, Y and U transcripts were similar over time.

**Figure 2:**
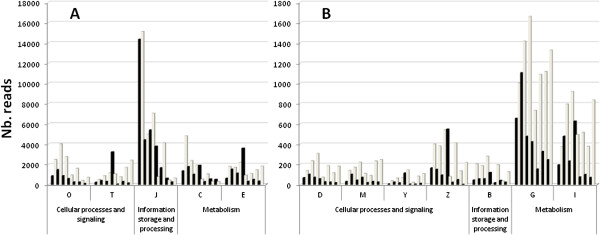
**Functional classification in yeast and mold genes expressed during Camembert cheese ripening.** Functional classification of clusters of euKaryotic Orthologous Groups (KOG database) in yeasts (black) and molds (grey) during Camembert cheese ripening. Scales were adjusted to fit categories with read numbers **(A)** generally over 1000 reads and **(B)** generally above 100 reads. Seven time points were taken (Day 5, 9, 15, 21, 35, 56 and 77), corresponding to key times in the ripening period. Read numbers were normalized to 100,000 reads/ripening day. The KOG categories presented belong to “Cellular processes and signaling” (D: cycle control, cell division, chromosome partitioning; M: Cell wall/membrane/envelope biogenesis; O: Post-translational modifications, protein turnover, chaperone functions; T: Signal transduction mechanisms; Y: Nuclear structures; Z: Cytoskeleton), “Information storage and processing” (B: Chromatin structures and dynamics; J: Translation); and “Metabolism” (C: Energy production and conversion; E: Amino acid transport and metabolism; G: Carbohydrate transport and metabolism; I: Lipid transport and metabolism).

Information on the metabolic pathways active in the transcriptome library was obtained from the crossed-analysis of the Gene Ontology (GO) annotation, Kyoto Encyclopedia of Genes and Genomes (KEGG) ontology (KO), and functional classification of clusters of euKaryotic Orthologous Groups (KOG database) [42-44]. The KOG database delivered the most informative analysis, providing 10% more affiliation of transcripts to a category than GO and KEGG [45,46]. Genes belonging to KOG categories D (Cell cycle control and mitosis), M (Cell wall/membrane/envelope biogenesis), Z (Cytoskeleton) and B (Chromatin structure and dynamics) were expressed at least 10-fold less than genes belonging to other KOG categories (Figure 2). Overall metatranscriptomic expression shows that, aside from translation (KOG category J; Figure 2A) and energy metabolism (KOG category C; Figure 2B), yeast transcripts dominated the early stages of ripening (day 5 and 9), while mold contigs experienced higher levels of expression around day 15. These transcription data matched the active growth phase of *G. candidum* and *P. camemberti*, as quantified by qPCR (Figure 1) [5,41,47-49].

### Central metabolism

**Figure 3:**
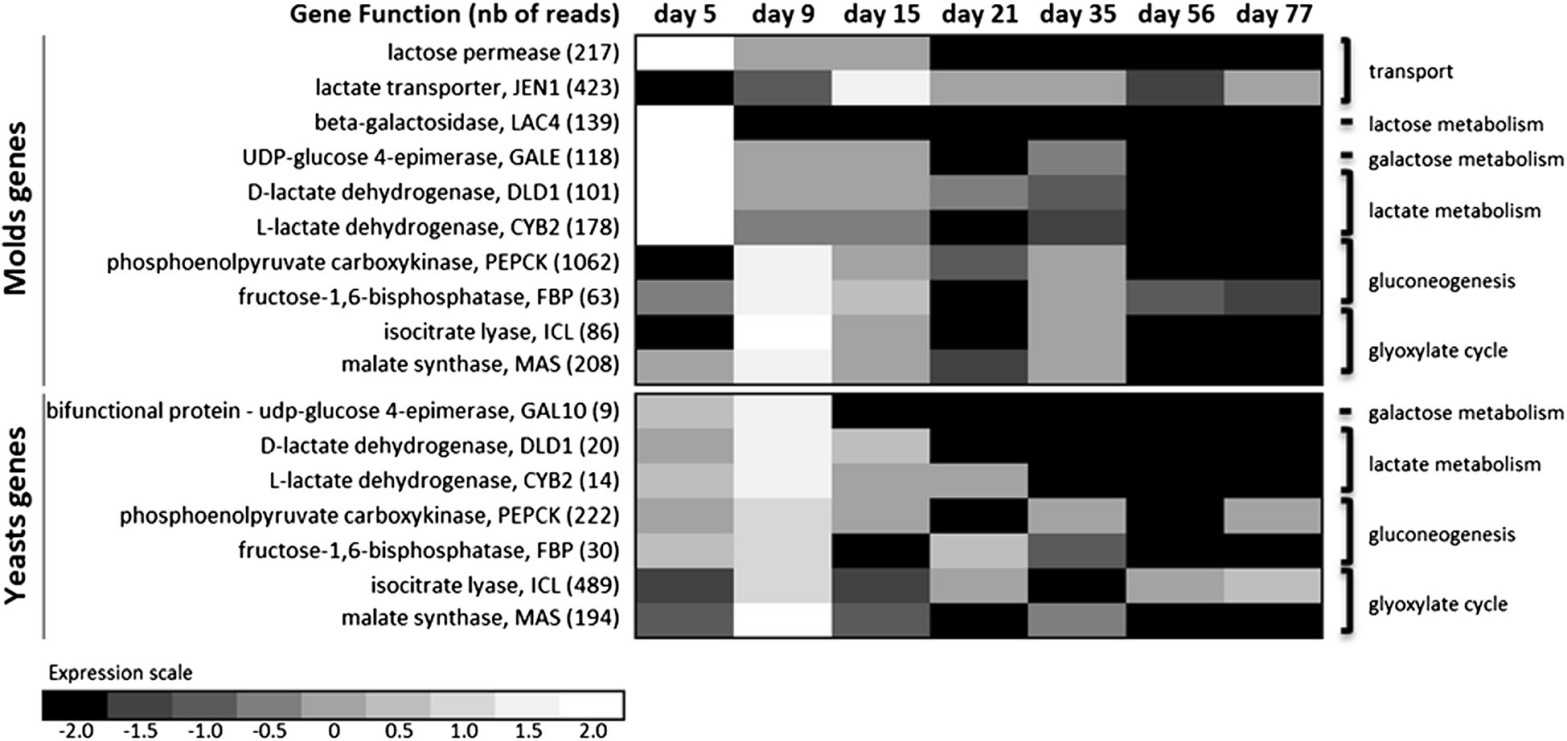
**Gene expression related to sugar and organic acid metabolism and transport.** For each gene function, total read number and relative expression during ripening are presented. On this heat map, relative expression is represented by a greyscale, between high (white) and low (black) expression levels.

According to KOG annotation, energy metabolism (KOG category C) was mainly expressed in the early stage of ripening (Figure 2). We identified numerous gene functions related to energy metabolism, including all enzymes in the glycolysis/gluconeogenesis, pentose phosphate (PP) pathways, tricarboxylic acid (TCA) cycle and oxidative phosphorylation, for both yeasts and molds. *P. camemberti* and *G. candidum* are, therefore, aerobic microorganisms capable of complete pyruvate degradation to CO_2_ and ATP production through carbohydrate, lipid and protein breakdown. For both fungal species, we identified 111 different contigs related to oxidative phosphorylation, including all five major complexes (NADH dehydrogenase, fumarate reductase, cytochrome bc1, cytochrome c oxidase and ATP synthase). Actually, energy metabolism was the dominant biological process in *CamemBank*01 (31% of all reads). Moreover, key enzymes in the glyoxylate bypass, namely isocitrate lyase (ICL; EC 4.1.3.1, respectively 489 and 86 reads found for yeasts and molds) and malate synthase (MAS; EC 2.3.3.9, respectively 194 and 208 reads), were found in high numbers [50]. In *CamemBank*01, most transcripts coding for those two enzymes are present at day 9 (Figure 3). Therefore, *P. camemberti* and *G. candidum* seem to be able to grow in a two-carbon source environment (acetate, ethanol, fatty acids), when other more complex carbon sources are unavailable [51,52].

### Lactose and lactate utilization in dairy *Fungi*

The presence of lactose and galactose influence microbial and fungal community development in the cheese matrix. Once β-galactosidase (LAC4, EC 3.2.1.23) hydrolyses lactose to form galactose and glucose, the latter is metabolized through the glycolysis, TCA cycle and PP pathways. Contigs related to lactose and galactose transport and utilization were expressed by molds only at the very beginning of cheese ripening (Figure 3), which is consistent with the negligible concentration of lactose in the rind after six days of ripening [5]. As expected, no evidence of lactose utilization was found in yeast contigs, confirming the well-known incapacity of *G. candidum* to assimilate lactose [53].

Lactate generated by lactic acid bacteria during cheese making is a major carbon source for surface fungal microflora in Camembert-type cheese. Its metabolism contributes to fungal growth and alkalinisation of the cheese surface [9]. For this purpose, a specific lactate transporter (JEN1) and two distinct lactate dehydrogenases, DLD1 (EC 1.1.2.4) and CYB2 (EC 1.1.2.3) [54,55], are essential. In *CamemBank*01, contigs coding for these enzymes were found for yeasts and molds (Figure 3). All non-fermentable carbon sources, such as lactate, are metabolized into sugars through the gluconeogenesis pathway and then redirected into central metabolism. Phosphoenolpyruvate kinase (PEPCK, EC 4.1.1.49) and fructose-1,6-bisphosphatase (FBP, EC 3.1.3.11) are two essential enzymes in this pathway. For yeasts and molds FBP and PEPCK are mainly expressed at days 9 and 15. PEPCK is massively expressed in both yeasts and molds, especially in the latter where it is among the top 1% of the most expressed contigs in *CamemBank*01 (Figure 3). This finding is consistent with the early expression of lactate metabolism related contigs, as well as ICL and MAS enzyme expression profiles in the early ripening stage, because of the possible depletion of glucose and lactose (Figure 3). At this stage, lactate, caseins and milk lipids are the dominant remaining energy sources [5] which explains the high transcription rate of the gluconeogenesis pathway. Considering its importance in fungal metabolism in relation to cheese production and its high expression in *CamemBank*01, the PEPCK transcript could be a useful biomarker to ensure the normal progression of the Camembert-type cheese ripening process.

### Protein metabolism

Proteolytic activity of fungal ripening cultures was proposed to be a key contributor to cheese flavor but only limited information is available. Analysis using the MEROPS peptidase database [56] (http://merops.sanger.ac.uk) identified 226 peptidases and five peptidase inhibitors in the *CamemBank*01 metatranscriptome. From this number, 89 (origin: 52 M; 19 Y; 18 U) were linked to the extracellular protein digestion category of the proteolysis activity. MEROPS analysis revealed that Metallopeptidase (MP) and Serine peptidases (SP) are the most abundant peptidase families expressed in yeasts and molds. Global expression profiles show that protease and peptidase transcripts are mainly detected in the first 21 days of the ripening period, supporting other findings indicating that proteolysis occurs mostly in the first two weeks of the ripening time [5,53,57,58].

**Figure 4:**
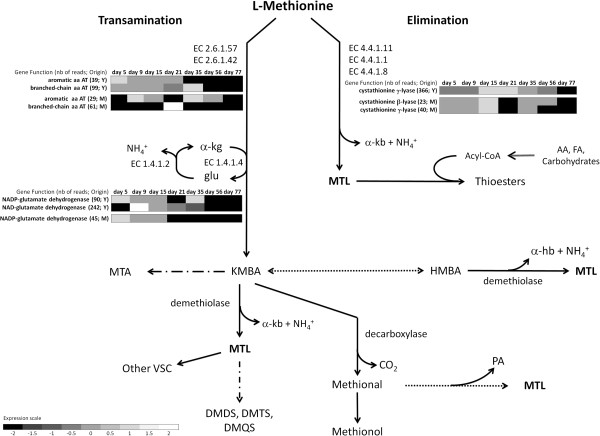
**Volatile sulfur compound formation through methionine catabolism.** Figure inspired from [67,68,76]. Expression data is represented by a greyscale, between high (white) and low (black) expression levels, for each enzymatic reaction. Legend: Y: Yeasts; M: Molds; AT: aminotransferase; α-kg: α-ketoglutarate; Glu: glutamate; gdh: glutamate deyhrogenase; α-kb: α-ketobutyrate; NH_4_^+^: ammonia; AA: aminoacids; FA: fatty acids; MTL: methanethiol; MTA: S-methylthioacetate; KMBA: 2-keto-4-methylthio butyric acid; HMBA: 4-methylthio hydroxybutyric acid; α-hb: α-hydroxybutyrate; VSC: volatile sulfur compounds; DMDS: dimethyl disulfide; DMTS: dimethyl trisulfide; DMQS: dimethyl tetrasulfide; PA: propionaldehyde. Solid lines: enzymatic reactions; double solid line: general metabolic pathways; simple dotted lines: potential enzymatic reactions; double dotted lines: chemical reactions.

In the cytoplasm, peptides and amino acids are catabolized by different enzymes that lead to the formation of aroma compounds [59-62]. Widely used ripening yeasts including *Kluyveromyces*, *Debaryomyces*, *Yarrowia* and *Geotrichum* are known for their volatile sulfur compound (VSC) biosynthesis through methionine degradation [63-66]. Most contigs involved in VSC production [11,12,67,68] were clustered in the KOG category E in *CamemBank*01 (Table 2, Figure 2). Methionine catabolism and the corresponding VSC production can occur in one (elimination pathway) or two steps (transamination or Ehrlich pathway) enzymatic reactions [69] (Figure 4).

**Table 2:** **Functional annotation statistics and expression data of contigs in *CamemBank*** **01**

**KOG category**	**General function**	**Yeasts [Y]**	**Molds [M]**
	**Metabolic pathway**	**(Nb contigs/Nb reads)**	
	**Metabolism and transport**		
**C**	Energy	122/9,155	182/14,014
**G**	Carbohydrates (sugars and organic acids)	58/4,141	152/9,778
**E**	Amino acids	102/10,139	183/12,707
**I**	Lipids	43/2,239	131/4,974
	**Cellular processes and signaling**		
**O**	Post-translational modifications	122/5,678	147/9,153
**T**	Signal transduction	55/5,952	168/4,859
	**Information storage and processing**		
**K**	Transcription	40/3,933	102/4,126
**J**	Translation	190/37,614	240/41,001
**A**	RNA processing and modification	35/1,250	136/3,787
	**Poorly characterized**		
**R**	General function prediction only	91/4,420	402/23,419
**S**	Unknown function or no annotation	184/9,664	1887/108,840

Cystathionine γ-lyase (CGL, EC 4.4.1.1) and cystathionine β-lyase (CBL, EC 4.4.1.8) (Figure 4) are two potential lyase candidates in the one-step generation of VSC through methionine catabolism [70]. In *CamemBank*01, *cgl* and *cbl* transcripts were found in molds, but only *cgl* transcripts were found in yeasts. The expression of both *cgl* and *cbl* was observed to be higher in yeasts throughout ripening (Figure 4). In *G. candidum*, *cgl* expression is linked to cabbage and sulfur aroma development in smear cheeses through methanethiol (MTL) production [71-73]. At an expression level of 366 reads, *cgl* is among the top 5% of expressed contigs in *CamemBank*01 and is a good candidate for producing the cabbage and sulphur notes *G. candidum* is known for [73]. These data suggest that *G. candidum* could be more involved in aroma and ammonia production through methionine catabolism than *P. camemberti*, considering that these enzymes are also linked to ammonia and α-ketobutyric acid production in *G. candidum*[74].

Transamination of methionine leading to MTL formation can be initiated by aminotansferases (Figure 4) [75]. In dairy *Fungi* the proposed pathway includes branched-chain (*BcAT*) and aromatic aminotransferases (*ArAT*) essential for flavor formation in *K. lactis*, *G. candidum* and *Yarrowia lipolytica*[63,64,66,76]. The next step of transamination is responsible for ammonia generation and is catalyzed by the NAD-glutamate dehydrogenase enzyme (NAD-GDH, EC 1.4.1.2) (Figure 4) [12,66]. In *CamemBank*01, *BcAT*, *ArAT* and *gdh* contigs were retrieved for yeasts and molds. The NAD-*gdh* contig was only found in yeasts (Figure 4). This observation confirms that *G. candidum* uses peptides and amino acids for energy metabolism and cellular growth, which contributes greatly to ammonia production and pH increase in cheese, while *P. camemberti* uses lactate [58,77-79]. According to the transcription data in *CamemBank*01, ammonia production and amino acid metabolism appear after the first week of ripening. Formation of α-keto-γ-methylthio butyric acid (KMBA) and MTL through the Ehrlich pathway may need an enzyme called KMBA demethiolase. Such a gene was not found in *CamemBank*01 and suggests, as others have previously stated, that the conversion of KMBA in MTL could be spontaneous and non-enzymatic [80,81]. In light of these observations, *CamemBank*01 outlines the need and provides the ability to investigate these metabolic pathways in depth, and to correlate these data with biochemical analysis.

### Lipid metabolism

Lipids have major roles in Camembert-type cheeses since they modulate the texture, act as the carrier for aroma compounds and are the major precursor for flavor compounds such as methylketones, lactones, esters and alcohols [2,62,82,83]. The lipid metabolism KOG category (I) is divided in two groups: fatty acid metabolism and cell wall-related lipid metabolism. Functional annotation of all contigs in *CamemBank*01 showed that fatty acid transport and metabolism counted for more than half of all of lipid metabolism (KOG I) contigs found in *CamemBank*01 (Table 2). Lipolysis pathways are expressed at the beginning of the ripening period; gene expression is limited at day 5 but increased at days 9 and 15 (Figure 2B). Seven transporters were also found, which had the same expression profile as all other lipid-related contigs.

Yeasts and molds that participate in the ripening of Camembert-type cheeses are known to possess lipases (EC 3.1.1.3) that hydrolyse triglycerides into di- and mono-glycerides, free fatty acids (FA) and glycerol. Only a few lipase transcripts were found in *CamemBank*01. According to GO annotation, all three lipases found have triglyceride lipase activity and, for *G. candidum*, two such enzymes were previously identified in the literature [84-88]. In both yeasts and molds, the contigs encoding lipase genes were expressed during the entire ripening period, but at a very low rate (under 71 reads/contig), which is consistent with the globally low expression of the lipolysis pathway genes compared to those of other metabolic pathways (Table 2).

**Figure 5:**
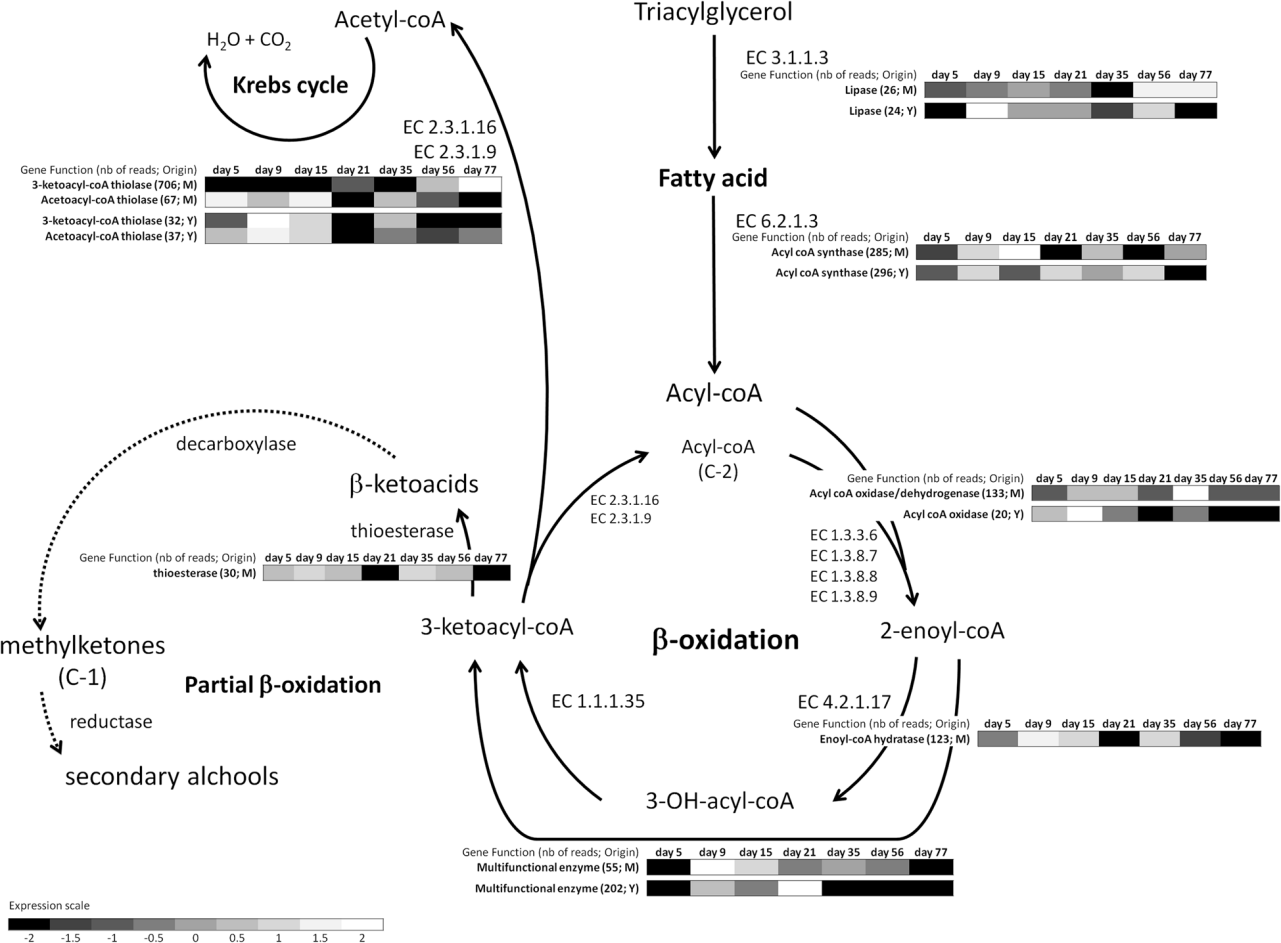
**Fatty acid metabolism and** β-**oxidation with associated expression data.** Figure adapted from [2,83]. Expression data is represented by a greyscale, between high (white) and low (black) expression levels, for each enzymatic reaction. Solid lines: enzymatic reactions; simple dotted lines: enzymatic reactions not found in *CamemBank*01.

Yeasts such as *Saccharomyces* and *Candida* appear to possess only the peroxisomal version of the β-oxidation pathway [89,90], while *Aspergillus* and *Podospora* possess both peroxisomal and mitochondrial pathways [91-93], consistent with *CamemBank*01 expression data. *CamemBank*01 expression data does not indicate the presence of a mitochondrial β-oxidation pathway in *G. candidum* but both pathways were identified in *P. camemberti*. Each cycle of β-oxidation produces one molecule of acetyl-coA that can be redirected into the TCA cycle to generate energy or transformed in ketone bodies (aroma precursors), and one molecule of acyl-coA that can go through other β-oxidation cycles (Figure 5). In *Fungi*, a peroxisomal multifunctional enzyme (MFE) is also responsible for the β-oxidation of fatty acids [91,94]. This enzyme combines the two middle steps (EC 4.2.1.17 and EC 1.1.1.35) of the β-oxidation cycle (Figure 5). In *CamemBank*01, we found the four enzymatic functions, including the MFE. The MFE’s expression profile is very different for yeasts and molds: in molds, it is expressed for most of the 2.5 month period of ripening, whereas in yeasts, it is clearly over-expressed at day 21 (Figure 5). Interestingly, 69% of all transcripts related to the β-oxidation cycle in yeast-related contigs coded for the MFE, suggesting that this enzyme could have a central biological role. In yeasts, MFE is the second most highly expressed of all lipolysis-related contigs, after the acyl-coA synthase (ACS, EC 6.2.1.3) (Figure 5). The acyl-coA synthase accounted for 44% of the total lipolysis-related transcripts. In molds, approximately 21% of transcripts coded for these two enzymes combined. From the perspective of finding potential biomarkers for Camembert-type cheese ripening, the multi-functional enzyme could be one of interest, given its expression over time in both microorganisms.

In the last degradation step of fatty acids, 3-ketoacyl-coA is redirected in the TCA cycle through a 3-ketoacyl-coA thiolase (KAT, EC 2.3.1.16) activity [95]. The high expression level in molds (706 reads in molds compared to 32 in yeasts) at the very end of the ripening period suggests that fatty acids are late energy sources for molds and that this gene could be an interesting biomarker to follow this activity. Finally, some fatty acids are only partially β-oxidized. Thioesterases, decarboxylases and reductases are then responsible for the potential production of methylketones and secondary alcohols, which are important aroma compounds in Camembert-type cheese [82]. During the ripening period of a Camembert-type cheese, fatty acids may be entirely degraded for energy production by *P. camemberti* and *G. candidum*. In fact, very few transcripts related to partial β-oxidation were found only in molds in *CamemBank*01, (30 reads total for a thioesterase gene; Figure 5). However, these findings confirm the hypothesis that *P. camemberti* has a higher lipolytic potential than *G. candidum* and its gene expression should be investigated more extensively [2].

## Conclusions

Overall, 7916 new contigs have been identified related to the metabolism of yeasts and molds that develop at the surface of a commercial Canadian Camembert-type cheese, increasing our knowledge about fungal metabolism. Considering that this cheese ecosystem was composed of two fungal strains, these data suggest that the transcripts associated with yeasts and molds potentially reflect the activity of *Geotrichum candidum* and *Penicillium camemberti. CamemBank*01 permitted us to simultaneously determine the sequence of a large part of the genetic information encoded by these two microorganisms and detail the expression of these putative genes. Since the previous genetic information available was mostly ribosomal DNA, *CamemBank*01 provides a data mining resource for the dairy *Fungi* scientific community. Whole genome sequencing improves knowledge of the genetic structure of an organism [96], while the comparison between genome sequences allows understanding the evolutionary structure of populations [14,16,97,98]. We demonstrated that NGS approach for transcriptome analysis is a powerful tool for acquiring massive genetic information in a given biological condition. Therefore, *CamemBank*01 can now contribute to the structural annotation of the genomic sequences of *P. camemberti* and *G. candidum*, when they will be available. Moreover, this new database has shown the genomic determinants responsible for the enzymatic and biochemical reactions occurring during soft cheese ripening, previously described by other authors. This metatranscriptome analysis helped to both demonstrate the presence and the expression of these genes in the cheese ripening process. Globally, for yeasts and molds, the same general functions (KOG categories C, G, E and I) seem to be participating in fungal metabolism during Camembert-type cheese ripening. These pathways are not only the most expressed in *CamemBank*01, but also the most relevant in terms of sensory properties. Selection and study of biological markers should be the next step in understanding the real contribution of individual fungal strains and the consortium. It is crucial to carry out a more in-depth study of their biochemical activity during cheese ripening, which will provide key information about their implication in the development of cheese flavor.

## Methods

### Cheese production and sampling

Commercial Camembert-type cheeses were provided by a producer of Canadian premium specialty cheeses. All cheeses were sampled from a regular production of an 870 g format pasteurized-milk Camembert from a high capacity cheesemaking facility for which the process is confidential. A commercial starter culture, containing thermophilic and mesophilic lactic acid bacteria, was used in combination with a ripening starter containing only *P. camemberti* LMA-1029 and *G. candidum* LMA-1028. Inoculation of the fungal strains provided an initial count of approximately 8 × 10^1^ CFU of *G. candidum* LMA-1028 and 6 × 10^3^ CFU of *P. camemberti* LMA-1029 per ml of milk. No other yeasts or molds were used as ripening agents to produce a cheese characterized by a mild proteolysis. Cheeses were ripened for the first 9 days at 13°C, 98% relative humidity, then wrapped and ripened at 4°C for up to 77 days. The total 77 day ripening period included the first 9 days prior to wrapping. Samples were analyzed at days 5, 9, 15, 21, 35, 56, and 77, which corresponds, chronologically to the appearance of the mycelium, through ripening, to the consumption period.

### DNA extraction and quantification of fungi

For each sampling time, mycelium from a 50 cm^2^ area (25 cm^2^ of both flat sides of the cheese) was recovered from cheese triplicates, frozen in liquid nitrogen and ground using a mortar and pestle. DNA extraction was performed according to Al-Samarrai *et al*. [99] using 20-25 mg of ground mycelium. Quantitative real-time PCR (qPCR) was performed as described in our previous work to detect and quantify two major fungal ripening cultures: *Geotrichum candidum* and *Penicillium camemberti*[41].

### RNA extraction, quality assessment and cDNA synthesis

To reduce possible sampling bias during the metatranscriptomic analysis, at each sampling time, the total RNA was extracted from three cheeses and each extraction was performed in triplicate. Total RNA was purified from 75 mg of frozen ground rind powder using the RNAqueous RNA isolation kit (Ambion) combined with the Plant RNA Isolation Aid solution (Ambion) in a 12:1 ratio, according to the manufacturer’s instructions. The quality of total RNA was evaluated using the RNA 6000 Nano Chip Kit (Agilent Technologies) and an Agilent 2100 Bioanalyzer (Agilent Technologies). For each sampling day, the three RNA extraction replicates were pooled at equal concentrations (1 μg/μL) and a 5 μL aliquot was incubated at 37°C for 2 h and analyzed again using the Agilent 2100 Bioanalyzer to ensure that no degradation had occurred. Reverse transcription was carried out using 1 μg of total RNA. cDNA was synthesized using the SMARTer PCR cDNA synthesis kit (Clontech) according to manufacturer’s instructions. Freshly synthesized cDNA samples were purified using the Wizard SV PCR Clean-up system (Promega) to remove residual nucleotides, enzymes and primers.

### Metatranscriptomic library preparation and cDNA sequencing

A metatranscriptomic library was created for each sampling day. Each library originated from three cheeses, from which RNA was extracted in three replicates, resulting in a pool of nine samples per ripening day. cDNA was fragmented using a Rapid library nebulizer (Roche/454 Sequencing) to obtain 750 bp fragments. The seven libraries (ripening days 5, 9, 15, 21, 35, 56, and 77) were prepared using the GS FLX Titanium Rapid Library preparation kit (454 Life Sciences). Each library was tagged with a unique barcode to be traced for analysis. Libraries were clonally amplified on beads by emulsion PCR using the GS FLX Titanium LV emPCR kit (454 Life Sciences). Beads with amplified libraries were loaded onto GS FLX Titanium PicoTiterPlate. Sequencing reactions were carried out using FLX Genome Sequencer (454 Life Sciences) with GS FLX Titanium reagents (454 Life Sciences). Data were initially processed using the GS Run processor software provided by 454 Life Sciences with default settings for image acquisition, base calling and quality estimation. Metatranscriptomic library synthesis and massive parallel sequencing was performed at Institut de Biologie Intégrative et des Systèmes (IBIS) at Université Laval (http://www.ibis.ulaval.ca/sequencage.shtml).

### Sequence assembly, mapping and quality assessment

A *de novo* assembly step was done using all 1,019,060 reads obtained from the seven time points and was named *CamemBank*01. This *de novo* assembly was performed using the gsAssembler module of Newbler (v2.5.3, 454 Life Sciences) with default parameters except for identity (95%) and overlapping length (40 nt). A trimming database was used to remove reverse transcription adapters (from SMARTer kit) from the sequencing reads prior to assembly. Newbler is an overlap-layout-consensus (OLC) assembler that merges short reads into non-redundant sequences without gaps (contigs) to obtain full transcript sequences. Data were manually filtered with the specific criteria of length (min. 200 nt) and read numbers were assigned to each contig (min. 6 assembled reads/contig), which reduced the number of contigs to 8,318. As an assembly validation step and to measure transcript numbers, we used the Newbler v2.5.3 gsMapper module (454 Life Sciences) to map individual sequencing reads back to the *de novo* database generated with gsAssembler, with an approach similar to what was done for the shrimp *Pandalus latirostris* transcriptome [100]. Default parameters were used for the mapping process except for 97.5% identity with existing contigs in *CamemBank*01, over a minimum of 20 nt. To remove assembly artefacts, such as redundancy, only the contigs showing 85- to 117% variation between number mapped and number assembled reads percentage were retained as high quality contigs in *CamemBank*01. This resulted in the exclusion of 402 contigs out of 8,318 (4.85%).

### Sequence identity and annotation

All 7,916 high quality contigs were submitted to automated Blastx annotation using Blast2GO software v2.5.0, with default parameters (e-value <0.0001) [42]. Subsequently, gene ontology (GO) was determined by using Blast2GO. Gene ontology terms corresponding to either one or all GO categories: biological processes (P), molecular functions (F) and cellular components (C), were assigned to each contig. This study focused on P and F categories because of their higher relevance in the description of fungal metabolism. Again, the annotation step was performed with default parameters except that the e-value parameter was set to <1e-6 to increase stringency. InterProScan analysis was performed with default parameters to find functional motifs, and then annotation refinement was performed with the Augment Annotation tool ANNEX in the Blast2GO software. Finally, Enzyme Code (EC) numbers were assigned.

A second annotation step was performed using a different database. The functional classification of clusters of euKaryotic Orthologous Groups (KOG database) [43] was preferred because it was globally more informative for *CamemBank*01. The NCBI KOG database containing 112,920 protein sequences from seven eukaryotic genomes was uploaded, and sequence comparison using Blastx against the database (e-value <0.0001) allowed the retrieval of KOG categories for each transcript. Data were sorted for each KOG group, at each day of ripening, for yeasts, molds and transcripts of uncharacterized origin. For this purpose, mapped reads were manually normalized to 100,000 reads per library.

Finally, nucleotide sequences of all 7,916 contigs were submitted to Kyoto Encyclopedia of Genes and Genomes (KEGG; http://www.genome.jp/kegg/kegg2.html) [101] through KEGG Automated Annotation Server tool (KAAS; http://www.genome.jp/tools/kaas/) for further functional annotation [102]. Using a single-directional best hit (SBH) blast method, KAAS compares nucleotide sequences to KEGG GENES database, allowing KEGG Orthology (KO) identifiers to be attributed to most contigs. Each contig with a KO identifier could then be mapped on KEGG metabolic pathways using KEGG mapper (http://www.genome.jp/kegg/mapper.html). Finally, we performed manual crossed-annotation using KOG, GO, KEGG databases and EC numbers.

### Sequence accession numbers

The initial reads data reported here have been submitted to NCBI sequence read archive (SRA, http://www.ncbi.nlm.nih.gov/sra) under accession number SRP030470. All contig sequences are available in the Transcriptome Shotgun Assembly Sequence database (TSA, http://www.ncbi.nlm.nih.gov/genbank/tsa). This TSA project has been deposited at DDBJ/EMBL/GenBank under the accession GAQB00000000. The version described in this paper is the first version, GAQB01000000.

### Semi-quantitative gene expression profiling in yeasts and molds for the identification of biological markers of the camembert cheese ripening period

Raw mapping data was used to visualize gene expression profiles during the ripening period. The fold change in expression for each transcript (Rx_i_) at each ripening time (y_i_) was calculated using a Serial Analysis of Gene Expression (SAGE) approach [103,104], according to the following formula (Eq. 1)

(1) $$\begin{array}{c} {\mathrm{Rx}}_{i}=\log_{2}\left[\left(n_{2}+f\right)/\left(n_{1}+f\right)\right]\\ \;+\log_{2}\left[\left(t_{1}-n_{1}+f\right)/\left(t_{2}-n_{2}+f\right)\right] \end{array}$$

where n_1_ is the average number of mapped reads for contig x_i_, n_2_, is the number of mapped reads for contig x_i_ at ripening day y_i_, t_1_ is the total of the average number of reads (sum of all x), t_2_ is the total number of reads at day y_i_ and f is the 0.5 correction factor.

## Competing interests

The authors declare that they have no competing interests.

## Authors’ contributions

MHL carried out all experiments and data analysis, except for cDNA sequencing. CV previously optimized RNA extractions and quality assessment protocols. BB was responsible for the preparation and sequencing of cDNA libraries and also contributed to data analysis. DSG and SL conceived of the study, and participated in its design and coordination. MHL, SL, BB and DSG draft the manuscript. All authors read and approved the final manuscript.
